# Supplementary material for: Developing novel antimicrobials by combining cancer chemotherapeutics with bacterial DNA repair inhibitors
Source: PLoS Pathog. 2023 Dec 7;19(12):e1011875. doi: 10.1371/journal.ppat.1011875 (PMC10729960; doi:10.1371/journal.ppat.1011875)
Supplement: S1 Table — (DOCX) [file ppat.1011875.s001.docx]

**S1 Table:** Calculated binding energies of compounds to UvrA’s ATP binding pockets and allosteric sites

| Compound | Binding energy (kcal/mol)* | | |
| --- | --- | --- | --- |
| ATP | -9.6 Distal | -9.2 Proximal |  |
| Pirarubicin | -9.8 Proximal | -9.6 Distal |  |
| Dienestrol | -8.7 Allosteric BP1 | -7.4 Distal |  |
| Mitoxantrone | -7.5 Proximal | -7.4 Distal |  |
| L-Thyroxine | -7.0 Allosteric BP2 | -6.9 Distal | -6.9 Proximal |

**Distal refers to C-terminal ATP binding pocket, proximal refers to N-terminal binding pocket, Allosteric BP (1,2) refer to newly determined allosteric sites located on the surface of UvrA (see Figure 4B).*
