# Supplementary material for: Developing novel antimicrobials by combining cancer chemotherapeutics with bacterial DNA repair inhibitors
Source: PLoS Pathog. 2023 Dec 7;19(12):e1011875. doi: 10.1371/journal.ppat.1011875 (PMC10729960; doi:10.1371/journal.ppat.1011875)
Supplement: S1 Fig — (DOCX) [file ppat.1011875.s002.docx]

**S1_Figure**

**UV damage repair assay controls**

**
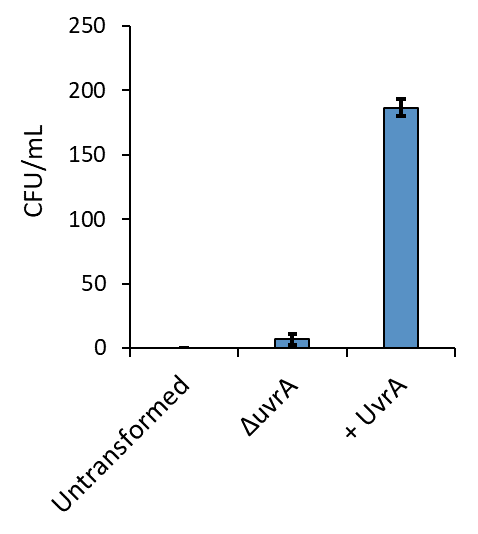
**

**Figure S1: UV damage repair assay controls.** The colony forming units (CFUs) of MG1655 spread on ampicillin plates in the absence of any introduced plasmid (untransformed), or when UvrA is knocked-out (*ΔuvrA*) show no growth. In the presence of functioning NER (+UvrA) the damage is efficiently repaired, conferring resistance, leading to substantial growth. Error bars represent the standard error of the mean (n≥3).
