# Supplementary material for: Developing novel antimicrobials by combining cancer chemotherapeutics with bacterial DNA repair inhibitors
Source: PLoS Pathog. 2023 Dec 7;19(12):e1011875. doi: 10.1371/journal.ppat.1011875 (PMC10729960; doi:10.1371/journal.ppat.1011875)
Supplement: S2 Fig — (DOCX) [file ppat.1011875.s003.docx]

**S2_Figure**

**
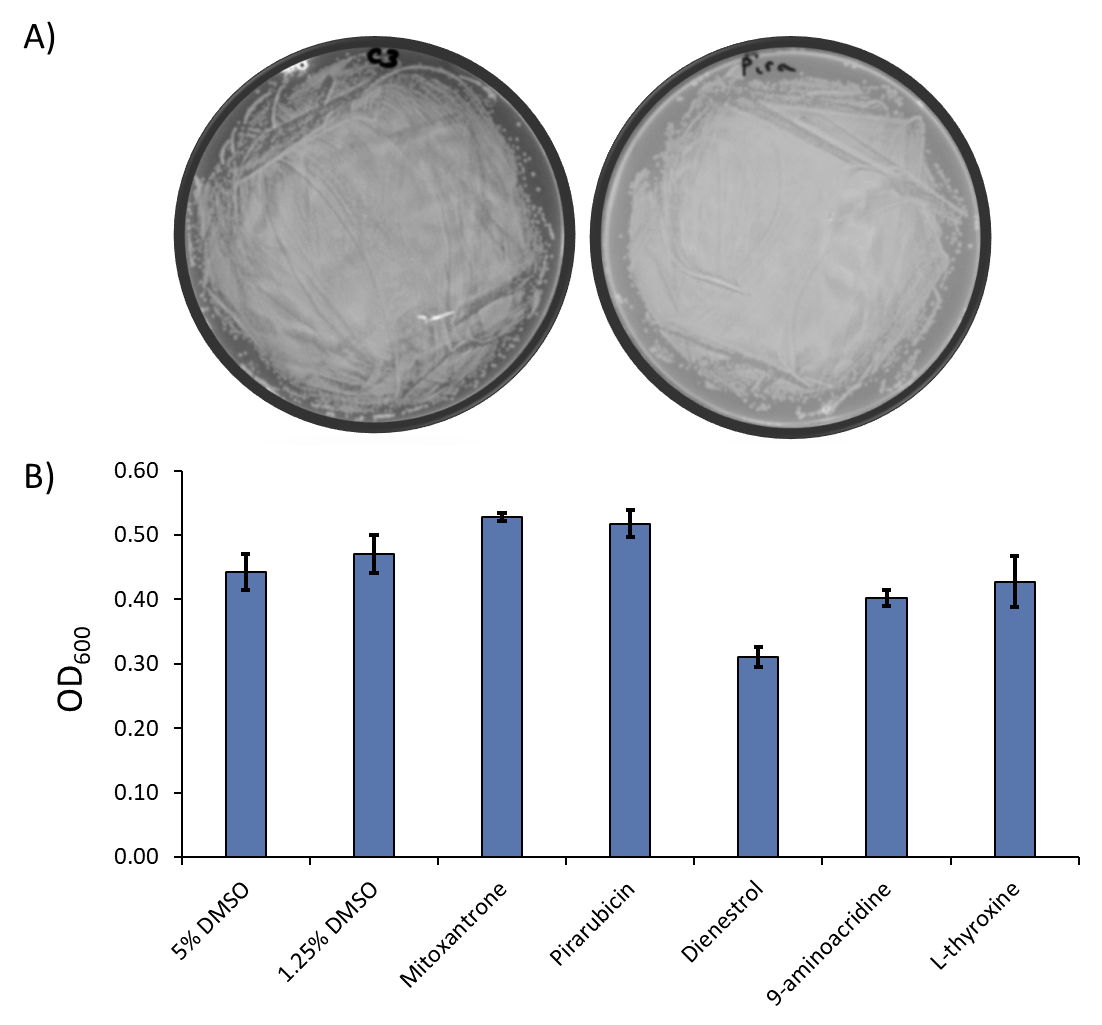
**

**S2 Figure: Compounds alone do not impair growth in the UV growth repair assay.** A) Example plate showing MG1655 spread on LB agar in the absence of added drug compounds (left) and in the presence of the same concentration of Pirarubicin used in figure 3D (right). No effect on growth is seen. The plates are representative of three different replicates for each of the compounds. B) OD_600_ of the same cultures shows a minimal decrease in cells density when incubated with the compounds. The largest effect measured of approximately 35% for Dienestrol is not large enough to explain the complete lack of growth in the UV damage repair assay.
