# Supplementary material for: Developing novel antimicrobials by combining cancer chemotherapeutics with bacterial DNA repair inhibitors
Source: PLoS Pathog. 2023 Dec 7;19(12):e1011875. doi: 10.1371/journal.ppat.1011875 (PMC10729960; doi:10.1371/journal.ppat.1011875)
Supplement: S3 Fig — (DOCX) [file ppat.1011875.s004.docx]

**S3_Figure**

**Intercalation controls**

**
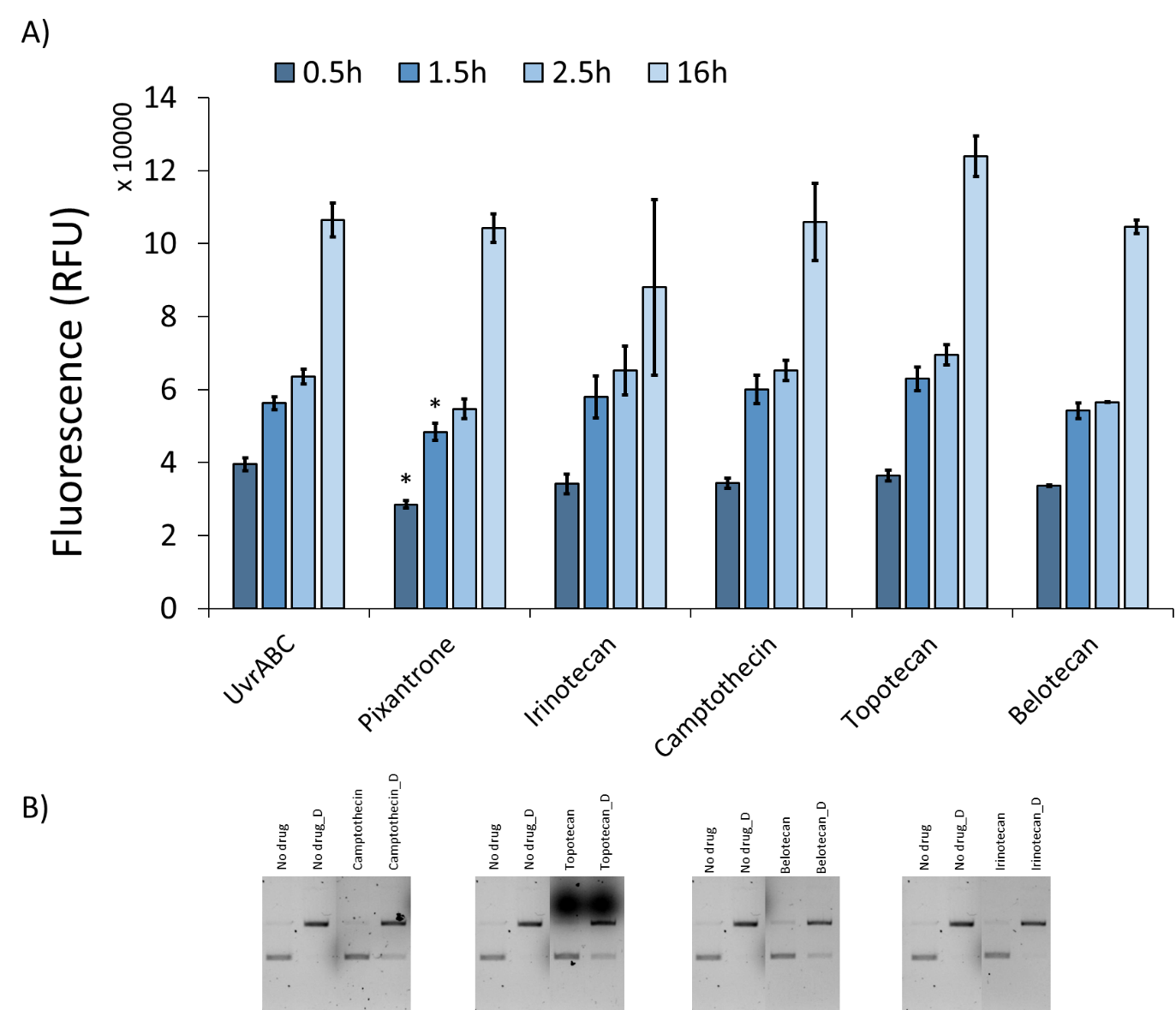
**

**S3 Figure: Known intercalators cannot inhibit the incision mediated by NER.** A) Known intercalators tested for their anti NER activity using the fluorescence incision assay. Pixantrone is a DNA intercalator [1] analogue to Mitoxantrone which resulted able to drastically inhibit incision at 16h. Pixantrone however is unable to considerably delay incision when tested in the fluorescence-base assay. The error bars represent the standard error of the mean (n≥2). The results were considered significant when compared to the untreated control (UvrABC) when p ≥ 0.05 (*). Irinotecan, Camptothecin, Topotecan and Belotecan are also known DNA intercalators [2,3] unable to significantly alter the incision when tested. B) Gel-based incision assay performed as further confirmation on the some of the compounds available. The results confirm what shown with the fluorescence-based incision assay, the intercalating action alone is not the primary responsible for UvrABC inhibition. The pictures are representative of two independent replicates.

**References**

1. Mukherji D, Pettengell R. Pixantrone for the treatment of aggressive non-Hodgkin lymphoma. Expert Opin Pharmacother. 2010;11: 1915–1923. doi:10.1517/14656566.2010.494180

2. Temerk Y, Ibrahim M, Ibrahim H, Schuhmann W. Comparative studies on the interaction of anticancer drug irinotecan with dsDNA and ssDNA. RSC Adv. 2018;8: 25387–25395. doi:10.1039/c8ra03231a

3. Staker BL, Hjerrild K, Feese MD, Behnke CA, Burgin AB Jr, Stewart L. The mechanism of topoisomerase I poisoning by a camptothecin analog. Proc Natl Acad Sci U S A. 2002;99: 15387–15392. doi:10.1073/pnas.242259599
