# Supplementary material for: Developing novel antimicrobials by combining cancer chemotherapeutics with bacterial DNA repair inhibitors
Source: PLoS Pathog. 2023 Dec 7;19(12):e1011875. doi: 10.1371/journal.ppat.1011875 (PMC10729960; doi:10.1371/journal.ppat.1011875)
Supplement: S4 Fig — (DOCX) [file ppat.1011875.s005.docx]

**S4_Figure**

**Cisplatin MIC against EC958 in the presence and absence of 2.5% DMSO**

**
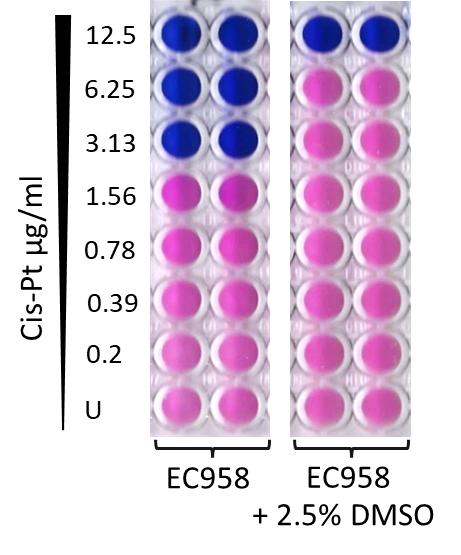
**

**S4 Figure: Cisplatin MIC in the presence and absence of 2.5% DMSO in EC958.** Colorimetric assay using the strongly coloured resazurin visualize cisplatin MIC in EC958. The assay shows a minimal inhibitory concentration of 3.13 µg/ml l and 12.5 µg/ml when respectively in absence and presence of 2.5% DMSO (first blue well indicating lack of growth).
