# Supplementary material for: Developing novel antimicrobials by combining cancer chemotherapeutics with bacterial DNA repair inhibitors
Source: PLoS Pathog. 2023 Dec 7;19(12):e1011875. doi: 10.1371/journal.ppat.1011875 (PMC10729960; doi:10.1371/journal.ppat.1011875)
Supplement: S5 Fig — (DOCX) [file ppat.1011875.s006.docx]

**S5_Figure**

**Antimicrobial effects of the combination between the lead compounds and the DNA damaging agent 4-NQO**

**S5 Figure: Inhibitory activity of selected hits against MG1655 in the presence and absence of 4-NQO.** Bar chart representation of the median fold decrease in MIC for MG1655 when the drug and cisplatin were combined (as shown in figure 4A). After inoculation at an initial OD_625_ of 0.001 the plates were incubated at 35 ± 2 ^o^C in a static incubator and protected from light for 18 h. At the end of the incubation 20 µl of buffered resazurin solution were added and the plate was imaged from the bottom after an ulterior incubation at 35 ± 2 ^o^C for 2 h. The major shifts were recorded and reported as the median fold decrease in MIC for both drugs. The FICi calculated showed a synergistic interaction for Mitoxantrone and 9-aminoacridine when combined with 4-NQO (FICi = 0.5) and partially synergistic for Pirarubicin (FICi = 0.625). The error bars represent the standard error of the mean (n≥3).
