# Supplementary material for: Developing novel antimicrobials by combining cancer chemotherapeutics with bacterial DNA repair inhibitors
Source: PLoS Pathog. 2023 Dec 7;19(12):e1011875. doi: 10.1371/journal.ppat.1011875 (PMC10729960; doi:10.1371/journal.ppat.1011875)
Supplement: Attachment — Submitted filename: Response to Reviewers.pdf [file ppat.1011875.s008.pdf]

We thank the editors and reviewers for taking the time to carefully examine our manuscript. There were a number of issues raised that we feel were mostly due to misinterpretations, to address this we have made changes to clarify our manuscript.

As mentioned by reviewer #1, the novelty of this manuscript lies in targeting bacterial UvrA that is structurally different from those in Human. Although, the authors present evidence that these drugs inhibit UvrA (E. coli), the first critical issue, as mentioned by reviewer #2, is the lack of results showing that human UvrA is unaffected by these drugs.

UvrA is only present among bacteria (and some archaebacteria) and completely absent in eukaryotic cells, including human cells. This is addressed in more detail in the response to reviewer #2. However, given that there are no homologous proteins in humans this is no longer a critical issue.

The second critical issue is that neither cisplatin nor UV radiation are specific to NER, as they also impact replication and recombination in E. coli. Therefore, given that the aim of this manuscript is to present the first steps towards the innovative idea of anti-NER treatments, it is pertinent to clarify this point. I found reviewer #2's proposal interesting to consider as an interesting complementary experiment to strengthen the data.

Contrary to the points made by reviewer #2, NER in bacteria is the primary repair mechanism for UV-induced damage in bacteria (this will be further discussed as a response to reviewer #2's comments). The downstream effects of unrepaired DNA would lead to consequences for replication and recombination. Secondly, it has been long established that NER is responsible for cisplatin adduct repair in *E. coli* (Beck 1985). We have also shown a sensitisation towards these genomic insults in a UvrA knockout construct (Bernacchia et al., 2022; Gupta et al., 2022). Overall, therefore, cisplatin and UV radiation are specifically repaired by NER.

The third critical issue is the lack of results arguing in support the results showing that the five drugs are lethal to E. coli without any treatment, since UvrA is not essential in E. coli.

It is not surprising to see cytotoxic effects of compounds when used at high concentrations; this is typical of many compounds that are not themselves useful as antimicrobials, due to off-target effects at these excessive concentrations. Our aim here is to simply reposition FDA approved drugs as sensitizers for the cytotoxic effects of cisplatin, and in the study, we titrate down their concentrations to that minimally required for sensitization.

In addition to the reviewers' comments, I would like you to pay attention to my minor comments I have listed below.

It should be of interest to discuss a little bit more the concept of anti-NER treatment as adjuvant. From my point of view, the adjuvants aim to make a normally resistant antimicrobial (with high MIC) effective (by decreasing the MIC) against a pathogen (here E. coli). However, here, the cisplatin concentration is necessarily fixed due to its use as an anti-cancer drug. What are you expecting : (i) anti-NER drug to be more efficient to clear infection or (ii) being efficient against cisplatin- resistant bacterial infections (if they exist)?

We thank the editor for requesting this greater explanation, which we have now included in the discussion (page 15). The argument we are making is that as correctly pointed out the maximum concentration of cisplatin is limited, and at this concentration it is below the MIC for *E. coli*. However, by using a second drug we sensitise the bacteria to cisplatin, lowering its effective MIC, and in this way meets the definition of an adjuvant. With respect to its mode of action we expect the adjuvant to clear

infection rather than targeting cisplatin resistance, for which we have no evidence of its existence. Indeed, our on-going study with patient derived samples might allow us to understand if cisplatin resistance exists, however this is out of the scope of the present study.

I encourage you to clarify whether you are expecting the anti-NER drugs to be bactericidal or bacteriostatic.

This is a good question, cisplatin has previously been found able to eradicate even persister cells (Chowdhury et al., 2016) suggesting a possibly strong bactericidal activity. We have added this statement to the manuscript in the discussion (page 15).

You should consider toning down the idea that targeting the NER could offer a new therapeutic approach. Indeed, it seems a bit of a stretch. In your study, anti-NER drugs decrease cisplatin concentrations that affect E. coli growth. So this only applies to cancer patients (treated with cisplatin) susceptible to superinfection.

Thank you for this suggestion, we agree and therefore in the revised manuscript we have toned this aspect and focused more on its application to cancer patients.

Please, justify why E. coli is a relevant pathogen for demonstrating the effect of anti-NER drugs on cancer patients. In cisplatin-treated cancer, is this pathogen most prevalent in event of infection?

As mentioned in the manuscript (and now expanded in the introduction, page 2), to address the relevance of our bacteria we used *E. coli* EC958 in addition to the reference MG1655 *E. coli* strain. EC958 has been found to be one of the major strains responsible for multidrug resistant urosepsis (Forde et al., 2014; Stocks et al., 2019; Totsika et al., 2011). Additional examples of the relevance of *E. coli* as an infectant in humans can be seen in two recent publications. Cassini and coworkers (Cassini et al., 2019) showed how antibiotic resistant *E. coli* are among the pathogens with the greatest clinical impact, supported by the ECDC observation (ECDC, 2019) that more than half of the *E. coli* isolates in EU are now resistant to at least one antimicrobial group.

Pay attention to reviewer #2's comment on figure 2C.

We have addressed the comments made by reviewer #1 about figure 2C (we presume that the reference made to reviewer #2 here was a typo, since no mention is made of Figure 2C by this reviewer).

Reviewer's Responses to Questions

Part I - Summary

Please use this section to discuss strengths/weaknesses of study, novelty/significance, general execution and scholarship.

Reviewer #1*: Bernacchia et al. present initial steps towards the innovative idea of targeting the bacterial nucleotide excision repair (NER) system in combination with chemotherapeutic treatment. Because chemotherapy typically also suppresses the patients' immune system, fighting infection in these patients by selectively killing pathogenic bacteria is a well-established and logical approach. The novelty of this manuscript lies in addressing prokaryotic NER, specifically the UvrA enzyme of prokaryotic NER. Conveniently, prokaryotic NER involves proteins, which are structurally completely different from those in eukaryotic NER, allowing the selective targeting of the bacterial DNA repair system (not the human). The authors present an optimised approach for screening for drugs (from an FDA approved compound library) that selectively target the bacterial NER system. Using a cell growth assay, they identified drugs that fulfil the requirement of being non-toxic for E. coli cells in the absence of additional cisplatin application as well as toxic in combination with cisplatin specifically under UV irradiation conditions (since the resulting UV lesions in DNA require repair by the NER system). The authors then tested the effects of the five most effective drug compounds on NER activity using a fluorescence based, spectroscopic in vitro DNA incision assay. They further determined (likely) binding positions of the compounds on UvrA via molecular docking and used a fluorescence-based UvrA ATPase activity assay as well as single molecule visualisation of DNA binding by UvrA to characterise the effects of these five best hits.*

I find this study very interesting and highly relevant (in light of growing antibiotic resistances). The manuscript is in general well written and the aim, approaches, and findings clearly presented. Nevertheless, I have a few minor issues with the manuscript as listed in detail below.

We thank the reviewer for their very well informed and positive assessment of our study. We outline our responses to the specific points below.

Reviewer #2*: The manuscript by Bernacchia outlines an approach to address bacterial infections during chemotherapy. This is a critical issue, given that many chemotherapeutic agents suppress the immune system. While the use of cisplatin is understandable for cancer therapy, it's not ideal for inhibiting NER.*

We thank the reviewer for recognising the importance of our objective. However, the reviewer is incorrect in their understanding of the role of cisplatin in this study, it was not used as an NER inhibitor but as a DNA damaging agent, against which drug discovery was used to find compounds that inhibit the subsequent DNA repair.

My primary concern is the specificity of the five drugs presented as NER inhibitors. Firstly, neither cisplatin nor UV radiation is specific to NER, as they also impact replication and recombination in E. coli. A drug like NQO or NFZ might offer a better starting point to demonstrate specificity.

We strongly disagree with the reviewer on this point, the very origin of the protein names found in NER derives from UV resistance. NER is the primary mechanism of repairing UV damage (Boyce & Howard-Flanders, 1964; Franklin & Haseltine, 1984; Pettijohn & Hanawalt, 1964; Sancar & Rupp, 1983; Setlow & Carrier, 1964; Thomas et al., 1985) and cisplatin induced damage (Beck et al., 1985; Husain et al., 1985; Popoff et al., 1987). With regards the second point, we recently published data using 4-

NQO in comparison to UV in uvra+ and uvra- *E. coli* strains(Bernacchia et al., 2022), these data are presented below:

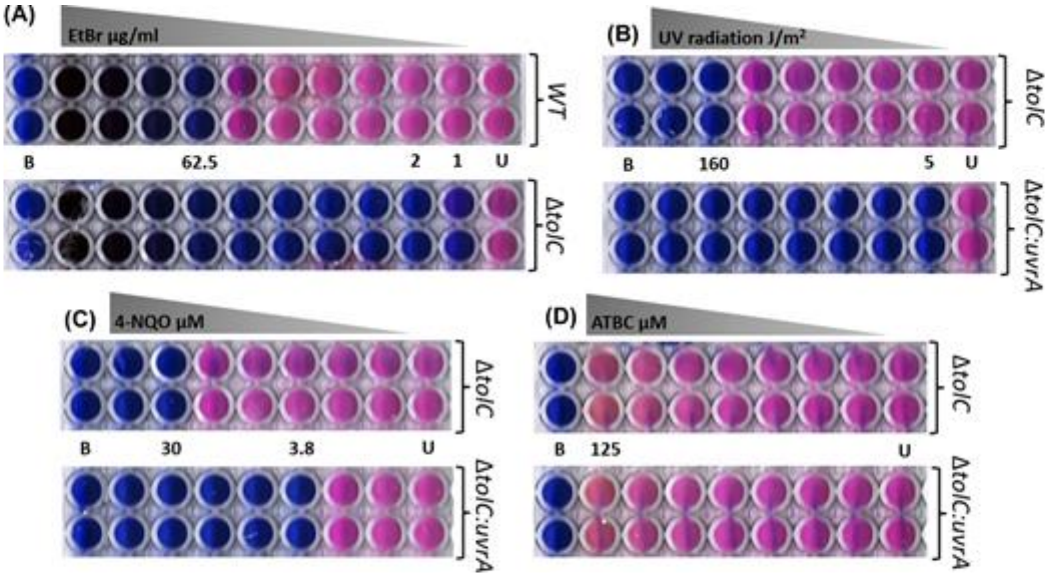

Figure 2 from Bernacchia et al., (2022) (https://doi.org/10.1042/BSR20220403)

It is clearly seen in B versus C that the change in MIC for a UvrA knockout strain versus WT is much greater when treated with UV versus 4-NQO, this indicates that UV offers a much more sensitive tool to study NER.

In their in vitro experiments, the authors present evidence that these drugs inhibit UvrA. However, essential controls appear to be missing. For instance, it would be crucial to show that human UvrA is unaffected by these drugs, or at a minimum, that other proteins with ATPase activity are not inhibited. While inhibiting an activity in vitro can be straightforward, demonstrating specificity can be more challenging.

As stated earlier, humans do not possess UvrA. There is a functional conservation of NER but no structural similarities exist (Petit & Sancar, 1999). Other (but not all) DNA repair pathways in bacteria do have human counterparts with structural conservation, which is why inhibiting NER is so attractive.

Lastly, I'm puzzled by the observation that their five drugs are lethal to E. coli even in the absence of any treatment, especially since UvrA is not essential in E. coli. This raises concerns about the specificity of the drugs in question.

Dosage is critical in considering drug toxicology; raising the concentration of any substance will affect cell viability for multiple diverse reasons. Our findings show an improvement in MIC when the drugs are combined versus single administrations. This enables us to titrate down the concentration of the FDA approved drugs, which would have unfortunate side-effects at the concentrations to which the reviewer refers. For drugs to impact multiple systems is extremely common (e.g., Aspirin (Miner & Hoffhines, 2007; Parvathaneni et al., 2019)), indeed it has been calculated that drugs affect ~2.5 different targets (Lu et al., 2012). The importance of any drug discovery study is then to optimize the interaction with the preferred target while minimizing any other effect. In summary, the aims of our study are not to propose drugs for immediate use in patients but to explore and evaluate a starting point for future investigations or chemical improvements by defining a novel approach. We have emphasized this point in the discussion (page 16 last paragraph) of the revised manuscript.

Reviewer #3: *I find that the manuscript presents a very important amount of encouraging findings. It is clearly written and is convincing.*

We thank this reviewer for their very positive endorsement of our work.

Reviewer #1: Methods

Is it common to present Materials & Methods details only in the Supplemental section in PLOS Pathogens?

We have revised the manuscript adding the material and methods in the main article.

Results

1) *Page 4 line 78: cisplatin concentration is given in units of µg/ml while all concentrations for the tested compounds are given in units of molarity throughout the manuscript. It might be easier for comparison to also give the cisplatin concentration in M.*

We have added the concentration in molar as suggested by the reviewer.

2) *Page 5 line 103: I think it is not entirely clear whether the five selected, best hits from the screening procedure came from the MG1655 strain or from the drug pump deficient MG1655 ΔtolC strain. It may be interesting to know whether drug efflux played a role and for which of the compounds. It is mentioned later in the manuscript (on page 10) that out of the five hits, Pirarubicin, Mitoxantrone, and 9-aminoacridine were identified from the wild type strain (with functional drug efflux pump). Two of these compounds are known to intercalate into DNA as stated by the authors, and the third (9-aminoacridine) has been shown, as mentioned by the authors in the Discussion, to modify DNA in combination with cisplatin. Is it possible that stable interactions with DNA by these three compounds are responsible for their retention in the cell in the wild type strain? Such DNA modification may also possibly prevent either binding to the DNA by UvrA or NER incision of the DNA, although the authors showed that other intercalators did not inhibit NER (Figure S3, see also point 9 below)? Is DNA binding by UvrA affected by 9-aminoacridine? The enhancement of ATPase activity in the presence of DNA seems only minor with this compound, while it is known that the ATPase activity of (wild type) UvrA is strongly enhanced in the presence of DNA (see point 7 below).*

We have added the clear information of the drug`s activity on the TolC strain as suggested by the reviewer to the results (page 5).

The reviewer suggests an intriguing mechanism for the retention of these compounds in WT MG1655 versus the TolC knockout. On the face of it our findings simply suggest that dienestrol and L-thyroxine are substrates of the efflux pump TolC and their active transport outside the cell lowers their concentration. As the reviewer correctly indicates UvrA's ATPase is stimulated by DNA therefore if 9-aminoacridine did affect UvrA's binding to DNA we would expect to see a reduction in ATPase activity in the presence of DNA; instead, we see the expected enhanced ATPase when DNA is present. This indicates 9-aminoacridine does not interfere with UvrA binding to DNA. It was for this reason we did not take 9-aminoacridine forward to the single molecule UvrA DNA displacement assay.

3) *Page 5 line 113/114: Why would incision of a single DNA strand 10 nt from the 3' end (with the*

attached fluorophore) result in "the 10 nt fragment leaving the duplex"? Is The DNA heated / what is the melting temperature?

We thank the reviewer for highlighting this omission, we have now expanded the explanation of this approach in the relevant sections of the material and methods. The experiment was carried out at 37°C, and the short 10 nt fragment formed subsequent to incision has a melting temperature of 16°C, explaining its release.

***4)** Page 6 last paragraph: include mentioning of Figures S1 and S2 and possibly also mention that drugs alone did not impair bacterial growth (Figure S2), since this seems like an important information.*

Thank you for this suggestion, we have incorporated this change (page 6).

5) Figure 2: (B) RFU not introduced; (D) CFU not introduced – only in Supplement; (C) arrows indicating which is the incision product would help here, it appears that the upper band is the incision product not the lower (usually the slower migrating species, i.e. the full length DNA not the incised, is shown at the top and the shorter DNA below); for L-thyroxin an incubation time of 15 minutes is stated (in contrast to the 30 minutes in (B)) – are all incubation times 15 minutes for all the compounds in (C)? It is surprising that the three assays, the fluorescence-based and gel-based incision assay and the in vivo UV recovery assay, give such different results for the five tested compounds. In all cases (or in most cases) all three assays confirmed suppression of NER activity, but the degree to which they did this was not consistent between the different assays (e.g. Dienestrol almost not significant in fluorescence-based and gel-based incision assay but best in in vivo assay). What could be reasons for this?

Thank you for bringing this up. We have addressed the comment by introducing RFU and CFU in the paper and further clarifying the incubation in the text and figure description (page 6 and Figure 2).

As the reviewer correctly states all three activity assays showed suppression of NER activity, the differences in magnitude are most likely due to the *in vivo* versus *in vitro* nature of the experiments. Retention of dienestrol within *E. coli* may have increased its local concentration thus increasing its effectiveness. Nonetheless, by using multiple assays we provide greater assurance that the effects are real, which would not be the case by using a single assay.

***6)** Page 7 mention Figure 3B (e.g. line 159)*

We have addressed the suggestions in the manuscript as suggested by the reviewer.

***7)** Figure 3: (A) I think it might be helpful to include UvrA without drug as a reference for the enhancement of ATPase activity by DNA (presumably the 100% is for UvrA without DNA). Also: what is the mechanism of NER inhibition by 9-aminoacridine, since ATPase activity by UvrA seems unaffected, at least in the absence of DNA. It also does not seem to bind to UvrA in the docking studies? See also point 2 above. (B) A colour legend would be helpful or, alternatively, the histogram bars in (C) could be colour-coded matching the colours of the different drugs in the zoom in (B).*

We apologise for the confusion, we have changed the figure description to clarify the meaning of the 100% line on the graph (legend Figure 3). This is actually the 100% value in each condition, so that each bar is expressed as a percentage change relative to the correct control. So, for 9-aminoacridine, we see the percentage activity unchanged in the presence of DNA and only slightly, but not significantly, reduced in the absence of DNA. The actual average enhancement of ATPase we observed was 56% upon the addition of DNA, which has now been added to the figure legend. We feel this data

presentation enables the reader to see the relative effects of drug without being overwhelmed by compensating for changes in ATPase as a consequence of DNA stimulation.

Since 9-aminoacridine was found unable to significantly affect UvrA in the ATPase activity both in the presence and absence of DNA, this suggests that the compound is able to target one of the other members of the system. We have made this argument in the discussion.

8) *Page 8 second paragraph: According to Figure 3B, L-Thyroxine and Dienestrol bind to allosteric sites at a small distance from the ATP binding sites in UvrA, while Mitoxantrone and Pirarubicin bind in the proximal ATP binding site of UvrA. From the ATPase assay it is not clear whether ATP binding or hydrolysis are affected for each of the five compounds. Is it only ATP hydrolysis that is reduced for Pirarubicin and Mitoxantrone or is it ATP binding per se, which may be partially blocked by the bound drugs. For the two drugs bound in the allosteric sites, L-Thyroxine and Dienestrol, single molecule fluorescence microscopy was carried out in addition, to observe effects on DNA binding by UvrA. The ATPase assay indicated that these two drugs had the strongest effect in suppressing UvrA's ATPase activity. For Dienestriol it appeared like the enhancement of ATPase activity in the presence of DNA was still present. While the single molecule visualisation is very cool, in principle a simple gel assay (EMSA) would have probably done the job. Here, while it is very clear that in the presence of Dienestriol or L-Thyroxine DNA binding by UvrA was strongly suppressed, the exact mechanism is not clear. Would an ATPase deficient mutant of UvrA bind to the DNA similar as wild type? Is DNA binding per se (in the absence of damage) not independent of UvrA ATPase activity? The authors have shown in a previous work that without ATP hydrolysis UvrA stays bound to DNA longer (Barnett and Kad, 2019, FASEB J). Or is DNA binding blocked by the bound drugs (which the ATPase data for Dienestriol would argue against)?*

These points raised by the reviewer are of great interest when it comes to teasing out the specific mechanism of inhibition. In this first of its kind study, it was our intention to highlight the principle of using NER inhibitors with cisplatin and we aim to address the precise mechanism in further studies beyond the scope of the present manuscript. However, with respect to our previous studies although we observed longer interactions of UvrA with DNA in the absence of ATP these interactions were much less frequent, therefore this is consistent with the reduced binding observed in presence of the newly discovered UvrA ATPase inhibitors (dienestrol and L-thyroxine) in this study. We have now added this to our amended manuscript (page 8 bottom).

9) *Page 8: Figure S3 (no NER inhibition by other intercalators) should be mentioned in results section before Figure S4 (e.g. lines 172-174).*

We apologise for this omission and have now addressed the suggestions in the manuscript made by the reviewer (page 6).

10) *The calculation of the FIC index may want to be included in the Methods section. Mention Figure 4B in the text (e.g. page 11 top)? Also, the effects of Pirarubicin, Mitoxantrone, and 9-aminoacridine, the three drugs tested on the multi-drug resistant strain EC958 as well as the MG1655 lab strain were rather different between the two strains. While they were all clearly effective, this raises the issue that their effects may vary drastically between different bacteria. Nevertheless, I believe this study is an excellent starting point for the exploration of the bacterial NER inhibition route in support of chemotherapy.*

We want to thank the reviewer for the positive comment. We have added the formula for the calculation of the FICi (in the relevant section in material and methods) as suggested and mentioned

the figure in the manuscript (page 12 top). We agree there may be different effects in different strains, and this forms the basis of an on-going study that we hope to report on when complete.

Reviewer #2: *(No Response)*

Reviewer #3: *Following a comment from previous reviewers, I think that the checkerboard assay is sufficient to show growth inhibition. As noted by one of the reviewers, if the authors want to conclude about killing or survival, then they would need to show a time kill assay (for the most promising condition for example). However, I think the checkerboard assay would be sufficient to show synergy between cisplatin and the tested drugs.*

We want to thank the reviewer for the positive feedback. To address this issue, we removed mention of killing versus survival from the manuscript and added the FICi data to discuss the degree of the cooperation between cisplatin and the drugs.

References

Beck, D. J., Popoff, S., Sancar, A., & Rupp, W. D. (1985). Reactions of the UVRABC excision nuclease with DNA damaged by diamminedichloroplatinum(II). *Nucleic Acids Research*, *13*(20), 7395–7412.

Bernacchia, L., Paris, A., Gupta, A. R., Moores, A. A., & Kad, N. M. (2022). Identification of the target and mode of action for the prokaryotic nucleotide excision repair inhibitor ATBC. *Bioscience Reports*, *42*(6). https://doi.org/10.1042/BSR20220403

Boyce, R. P., & Howard-Flanders, P. (1964). Release of ultraviolet light-induced thymine dimers from DNA in E. coli k-12. *Proceedings of the National Academy of Sciences of the United States of America*, *51*(2), 293–300.

Cassini, A., Högberg, L. D., Plachouras, D., Quattrocchi, A., Hoxha, A., Simonsen, G. S., Colomb-Cotinat, M., Kretzschmar, M. E., Devleesschauwer, B., Cecchini, M., Ouakrim, D. A., Oliveira, T. C., Struelens, M. J., Suetens, C., Monnet, D. L., Strauss, R., Mertens, K., Struyf, T., Catry, B., … Hopkins, S. (2019). Attributable deaths and disability-adjusted life-years caused by infections with antibiotic-resistant bacteria in the EU and the European Economic Area in 2015: a population-level modelling analysis. *The Lancet Infectious Diseases*, *19*(1), 56–66.

Chowdhury, N., Wood, T. L., Martínez-Vázquez, M., García-Contreras, R., & Wood, T. K. (2016). DNA-crosslinker cisplatin eradicates bacterial persister cells. *Biotechnology and Bioengineering*, *113*(9), 1984–1992.

ECDC. (2019). *Surveillance of Antimicrobial Resistance in Europe: Annual Report of the European Antimicrobial Resistance Surveillance Network (EARS-Net) 2018*. European Centre for Disease Prevention and control. https://doi.org/10.2900/22212

Forde, B. M., Ben Zakour, N. L., Stanton-Cook, M., Phan, M.-D., Totsika, M., Peters, K. M., Chan, K. G., Schembri, M. A., Upton, M., & Beatson, S. A. (2014). The complete genome sequence of Escherichia coli EC958: a high quality reference sequence for the globally disseminated multidrug resistant E. coli O25b:H4-ST131 clone. *PloS One*, *9*(8), e104400.

Franklin, W. A., & Haseltine, W. A. (1984). Removal of UV light-induced pyrimidine-pyrimidone(6-4) products from Escherichia coli DNA requires the uvrA, uvrB, and urvC gene products. *Proceedings of the National Academy of Sciences of the United States of America*, *81*(12), 3821–3824.

Gupta, A., Bernacchia, L., & Kad, N. M. (2022). Culture media, DMSO and efflux affect the antibacterial activity of cisplatin and oxaliplatin. *Letters in Applied Microbiology*. https://doi.org/10.1111/lam.13767

Husain, I., Chaney, S. G., & Sancar, A. (1985). Repair of cis-platinum-DNA adducts by ABC excinuclease in vivo and in vitro. *Journal of Bacteriology*, *163*(3), 817–823.

Lu, J.-J., Pan, W., Hu, Y.-J., & Wang, Y.-T. (2012). Multi-target drugs: the trend of drug research and development. *PloS One*, *7*(6), e40262.

Miner, J., & Hoffhines, A. (2007). The Discovery of Aspirin's Antithrombotic Effects. *Texas Heart Institute Journal / from the Texas Heart Institute of St. Luke's Episcopal Hospital, Texas Children's Hospital*, *34*(2), 179.

Parvathaneni, V., Kulkarni, N. S., Muth, A., & Gupta, V. (2019). Drug repurposing: a promising tool to accelerate the drug discovery process. *Drug Discovery Today*, *24*(10), 2076–2085.

Petit, C., & Sancar, A. (1999). Nucleotide excision repair: from E. coli to man. *Biochimie*, *81*(1–2), 15–25.

Pettijohn, D., & Hanawalt, P. (1964). Evidence for repair-replication of ultraviolet damaged DNA in bacteria. *Journal of Molecular Biology*, *9*(2), 395–410.

Popoff, S. C., Beck, D. J., & Rupp, W. D. (1987). Repair of plasmid DNA damaged in vitro with cis- or trans-diamminedichloroplatinum(II) in Escherichia coli. *Mutation Research*, *183*(2), 129–137.

Sancar, A., & Rupp, W. D. (1983). A novel repair enzyme: UVRABC excision nuclease of Escherichia coli cuts a DNA strand on both sides of the damaged region. *Cell*, *33*(1), 249–260.

Setlow, R. B., & Carrier, W. L. (1964). The disappearance of thymine dimers from DNA: an error-correcting mechanism. *Proceedings of the National Academy of Sciences of the United States of America*, *51*(2), 226–231.

Stocks, C. J., Phan, M.-D., Achard, M. E. S., Nhu, N. T. K., Condon, N. D., Gawthorne, J. A., Lo, A. W., Peters, K. M., McEwan, A. G., Kapetanovic, R., Schembri, M. A., & Sweet, M. J. (2019). Uropathogenic *Escherichia coli* employs both evasion and resistance to subvert innate immune-mediated zinc toxicity for dissemination. *Proceedings of the National Academy of Sciences of the United States of America*, *116*(13), 6341–6350.

Thomas, D. C., Levy, M., & Sancar, A. (1985). Amplification and purification of UvrA, UvrB, and UvrC proteins of Escherichia coli. *The Journal of Biological Chemistry*, *260*(17), 9875–9883.

Totsika, M., Beatson, S. A., Sarkar, S., Phan, M.-D., Petty, N. K., Bachmann, N., Szubert, M., Sidjabat, H. E., Paterson, D. L., Upton, M., & Schembri, M. A. (2011). Insights into a multidrug resistant Escherichia coli pathogen of the globally disseminated ST131 lineage: genome analysis and virulence mechanisms. *PloS One*, *6*(10), e26578.
