# Supplementary material for: Developing novel antimicrobials by combining cancer chemotherapeutics with bacterial DNA repair inhibitors
Source: PLoS Pathog. 2023 Dec 7;19(12):e1011875. doi: 10.1371/journal.ppat.1011875 (PMC10729960; doi:10.1371/journal.ppat.1011875)
Supplement: Attachment — Submitted filename: Reviewer Response.docx [file ppat.1011875.s007.docx]

We thank the reviewers for taking the time to critique our manuscript. As a result, we have made substantial changes throughout the manuscript and feel it is much better as a result. All of the changes are detailed below.

Reviewer #1 expertise: Cancer treatments

Reviewer #2 expertise: Antibacterial drugs combination

***Reviewer #1:*** *There is a fair amount of existing literature on adjuvant antimicrobial therapy in cancer treatment and I found this manuscript insufficient in placing its results in the context of existing work. Providing more of this context could better motivate the authors' very specific focus on cisplatin and targeting NER in terms of clinical cancer treatment. Overall, I found this manuscript somewhat interesting but would have appreciated further investigation into the actual feasibility of using the identified drugs as adjuvant therapy for cisplatin treatment -- for example, what is the impact of combination cisplatin and antimicrobial drugs (at the dosages suggested for E coli) on cancer cells, and have toxicity profiles in combination been considered? Are other antimicrobial targets more effective in controlling cancer-associated pathogen infections, and do these vary between anti-cancer therapies?*

We agree with the reviewer that adjuvant antimicrobials are not novel, for example clavulanic acid is used with amoxicillin (Hunter et al. 1980). **However, to our knowledge no combination of a compound that targets nucleotide excision repair in bacteria to halt replication when exposed with cancer chemotherapeutics has previously been made.** The approaches we outlined in our manuscript have opened the door for the development of novel tools to tackle infections in patients undergoing cancer chemotherapy. As a result, our identified compounds are currently undergoing a pre-clinical trial to determine their efficacy with samples collected from febrile patients undergoing chemotherapy.

Furthermore, this study is the first step in the design of specific inhibitors based on the previously unidentified allosteric binding pockets we identified. Altogether, our message is that this is the first step for the discovery/design of new potent inhibitors as we now state in the discussion. The additional experiments suggested would be extremely useful for further development but would take months to years to complete and go beyond the scope of this work.

*Minor comments:*

*Line 119: should inside the parentheses say "from top to bottom"?*

The figure has been modified and the text re-written.

***Reviewer #2:*** *In the manuscript "Combining cancer chemotherapeutics with bacterial DNA repair inhibitors to develop novel antimicrobials" Bernacchia et al. screened for potential antimicrobial compounds inhibiting the growth of E. coli in the presence of a chemotherapeutic agent called cisplatin. As the high rate of lethal bacterial infections in cancer patients is a problem of ever-increasing importance, new approaches to fight against these infections are of great necessity. Cisplatin inhibits cell division in both bacteria and in human cells by stalling replication through the formation of DNA adducts with inter- and intra- strand crosslinks that can be repaired by enzymes in the nucleotide excision DNA repair pathway (NER). The authors aimed to find potentially novel compounds that might act as antimicrobials in combination with the cisplatin by specifically targeting the bacterial NER pathway (that has very little homology to its human counterparts). Therefore, with impaired NER, bacteria would not be able to repair the damage caused by cisplatin during cancer chemotherapy. In this study, they identified a series of NER inhibitors by screening a library of FDA-approved compounds that inhibit the growth of E. coli at a dosage of 20uM only in the presence of a sub-lethal dose of cisplatin. They have also confirmed the mechanism of action for five available candidates as inhibition of NER by testing for DNA incision in vitro and in vivo, using in silico docking and single molecule assays. Overall, the study raises very important new aspects of infection treatment strategies for cancer patients by applying newly discovered compounds which antimicrobial effect can be significantly enhanced by cisplatin-based cancer chemotherapy. However, there are statements in the manuscript that are a bit vague and there is a lack of quantitative definition of drug interaction (such as additive or synergistic drug interactions). Without this it is hard to judge whether some of their newly discovered compounds may confer bacterial inhibition independent of the effect of the cisplatin (showing additive rather than synergistic effect).*

*Major points:*

*In the introduction (line# 48-50) they claim that "Inhibition of NER alone does not kill bacteria (19, 20), therefore, in this study, we identified a series of NER inhibitors by screening a library of FDA-approved compounds that kill E. coli only in the presence of a sub-lethal dose of cisplatin". First, bacterial growth inhibition was measured and not bacterial killing, second, each of these newly discovered antimicrobials as well as the cisplatin inhibited the growth of the E. coli alone (shown on the checkerboard plates). Therefore, a more proper statement would be that sublethal level of cisplatin decreased the MIC of these compounds below 20uM. The same applies to the statement in the discussion (line 253-255): "In this study we have developed an approach to the discovery and testing of adjuvant antimicrobials that only possess antibacterial activity in the presence of the cancer chemotherapy drug cisplatin."*

*The authors state throughout the text that their candidate combinations (found in the colorimetric resazurin assay) increased bacterial killing or decreased bacterial viability or survival. I would suggest removing the words "kill", "viability" or "survival" and they should call the measured phenotype everywhere throughout the text as growth inhibition as it was done in the title of their result section ("Screening for growth inhibitors in E. coli").*

We thank the reviewer for pointing out the importance of the language used, for which we agree, and therefore have changed all statements to reflect the actual effects of the drugs as suggested.

*The proper quantitative evaluation of the drug interaction scores is missing. This score is calculated using the isobole lines of the 2D response surface (measured on checkerboard plates) rather than plotting the MIC decrease on an arbitrary chosen single concentration of the drugs. It can be easily the case that most of the combinations have an overall additive effect on the bacterial growth inhibition, thus suggesting that the NER inhibitors may confer bacterial inhibition independent of the effect of the cisplatin. According to the hypothesis of the authors that the NER inhibitors act in cooperation with the cisplatin there should be a strong synergy between these newly discovered compounds and the cisplatin.*

We thank the reviewer for their thoughts on this and have addressed this by estimating the synergy using the FIC index. In addition, we discuss the limited relevancy of such an approach since in this study we are not looking for the optimal combination of both drugs since the amount of cisplatin is necessarily fixed due to its use as an anti-cancer drug. Changes have been made throughout the manuscript.

*It would be important if an additional negative control were included in the experiments of Figure 3, for example a drug like cisplatin that is not expected to inhibit nucleotide excision repair.*

We have included additional compounds in supplementary figure S3. This shows anticancer compounds that are unable to significantly inhibit incision, including an mitoxantrone analogue.

*Authors have tested the FDA approved drug library on both a TolC-knockout and a wild type MG1655 strain. However, they have not summarized the list of the drugs with increased efficacy in the presence of the cisplatin on both strains, only on the TolC-knockout or only on the WT-MG1655 strain (on Figure 1). It would be important to also include those hits as potentially relevant which were only effective against the WT strain and not against the TolC-knockout. As the loss of TolC is also known to increase resistance of bacteria to some antibiotics, which use outer membrane porins as entry routes into the bacterial cell.*

Our selected compounds were the best performers across the range of selection criteria that we used (as highlighted in the flowchart in figure 1). Those compounds that were effective across both TolC and MG1655 are shown, however we do not include the many hits against MG1655 because each were rejected for numerous reasons, and some were not progressed through all tests as a result. Therefore, we have not included these additional hits for that reason.

*Minor points*

*How the authors explain the differences on the observed drug interactions between the 3 NER inhibitors and cisplatin on the lab E. coli (MG1655) and on the clinical strains? It looks like that the 9-aminoacridine in combination with the cisplatin hardly show any synergism against the clinical isolate however this combination was the strongest hit against the TolC-knockout and MG1655 strains.*

*Were the MICs for both the cisplatin and NER inhibitors identical among the TolC-knockout, WT-MG1655 and the clinical isolate? Increased MIC of the NER inhibitors against the clinical isolate compared to the MG1655 might indicate that the compounds can be efficiently removed by drug efflux. Is this tested clinical isolate (EC958) known to carry mutations affecting drug efflux?*

The TolC data is not clinically relevant and only used to identify hits, therefore to remove any confusion we felt it appropriate to remove further analysis of TolC from the study. This permits the paper to focus on the two more relevant strains, wild-type and EC958, which exhibit a similar inhibition profile. 9-aminoacridine showed the same MIC across the strains used while pirarubicin and mitoxantrone had a higher MIC in EC958 and the wild-type strain compared to the TolC mutant, indicating a role of the efflux pump in the execration of these compounds.

**References**

Hunter, P. A., K. Coleman, J. Fisher, and D. Taylor. 1980. “In Vitro Synergistic Properties of Clavulanic Acid, with Ampicillin, Amoxycillin and Ticarcillin.” The Journal of Antimicrobial Chemotherapy 6 (4): 455–70.
