# Supplementary material for: Developing novel antimicrobials by combining cancer chemotherapeutics with bacterial DNA repair inhibitors
Source: PLoS Pathog. 2023 Dec 7;19(12):e1011875. doi: 10.1371/journal.ppat.1011875 (PMC10729960; doi:10.1371/journal.ppat.1011875)
Supplement: Attachment — Submitted filename: Screening Editor final response_final.docx [file ppat.1011875.s009.docx]

Please find below our responses to both Prof Guillard’s comments and those of the reviewers. We have performed extra experiments and made editorial changes to the manuscript to deal with the issues raised.

**Editor’s comments:**

1) Assess NER inhibitors upon exposure to ciprofloxacin to answer to reviewer#2.

Fluoroquinolones (among which ciprofloxacin) have been shown to cause DNA damage, activate the SOS response system and can even cause the formation of CPDs (Bhattacharya, Mukherjee, and Mandal 2020; Merschsundermann et al. 1994). These effects would create lesions that require nucleotide excision repair (McDaniel, Rogers, and Hill 1978; Mok and Brynildsen 2018) and while very interesting, these interactions would be difficult to interpret and beyond the scope of our study. Therefore, we went back to reviewer#2’s suggestion to examine the interaction with 4NQO. These new experiments confirmed our previous results that there is a combinatorial effect of the lead compounds and the DNA damaging agent. We present these data in the supplementary material (Figure S5) and have also included a statement referring to these new results in the main manuscript (page 12).

2) Please tone down the specificity of your drugs towards the NER system by mentioning that UV/cisplatin-induced lesions block DNA replication.

Following the suggestion, we have now mentioned that UV stalls replication (page 5). Moreover, we had already included in pages 1 & 2 multiple mentions of cisplatin’s ability to block replication.

3) Clearly state that you have not assessed whether your drugs had no impact on the human homolog of UvrA. However, (i) the closest sequence homolog (25% homology) of uvrA is an ABC transporter of insulin due to its ATPase domain (see reference provided by reviewer#3) and (ii) the drugs used here are FDA approved (I agree with reviewer#3), therefore, we can anticipate no innocuity for human and therefore no effect on potential human NER proteins or any other ATPase proteins.

Following the suggestion, we have now clarified that UvrA has no human homologues including a statement on the closest sequence homology found in homo sapiens. Furthermore, we have clearly mentioned that we did not perform tests with eukaryotic cells or proteins. These edits can be found in page 14.

4) Provide data (from literature, database…) indicating that E. coli is mainly found in infections occurring in cisplatin-treated cancer patients. Indeed, your reply to my previous comment merely explains that urosepsis is due to resistant E. coli. It is worth justifying your assay with E. coli and not another pathogen in the clinical context of the patients you are targeting.

We have clarified the choice of *E. coli* by expanding on our existing justifications. Amongst the most common infectants during chemotherapy-induced neutropenia have been reported to be gram negative bacteria including *E. coli* (Crawford, Dale, and Lyman 2004). Furthermore, *E. coli* has been directly isolated from patients with neutropenia receiving cisplatin treatment for cancer (Curtin et al. 1991). We have now included a direct mention of this relevance in the manuscript. The edit can be found in pages 2 & 3.

**Reviewer’s comments:**

Reviewer #1: All of my comments have been adequately addressed by the authors.

We want to thank the reviewer for their positive endorsement of our work.

Reviewer #2: 1. We strongly disagree with the reviewer on this point, the very origin of the protein names found in NER derives from UV resistance. NER is the primary mechanism of repairing UV damage (Boyce & Howard-Flanders, 1964; Franklin & Haseltine, 1984; Pettijohn & Hanawalt, 1964; Sancar & Rupp, 1983; Setlow & Carrier, 1964; Thomas et al., 1985) and cisplatin induced damage (Beck et al., 1985; Husain et al., 1985; Popoff et al., 1987). With regards the second point, we recently published data using 4- NQO in comparison to UV in uvra+ and uvra- E. coli strains(Bernacchia et al., 2022), these data are presented below.

I strongly disagree with the authors regarding the specificity of UV and cisplatin. Citing papers from the last century does not strengthen their case. While they are correct that UV/cisplatin lesions are removed by NER, these lesions also block DNA replication. For instance, a recF mutant is sensitive to both drugs and is not involved in NER (see Justin Courcelle, David J. Crowley, and Philip C. Hanawalt, J. Bact. 1999).

The involvement of recombination repair is indeed an interesting point that we have now addressed by expanding the Discussion to include the observation on the possible role of recombination repair in relation to nucleotide excision repair (pages 14-15).

Furthermore, their argument about the greater change in MIC for a UvrA knockout strain versus WT when treated with UV as opposed to 4-NQO is flawed. These are entirely different treatments, and their specificity can't be compared. It seems conducting the NQO experiments would be simple, cost-effective, and provide a clear answer. I am curious about how these new NER inhibitors will behave in the presence of Cipro, which doesn't require NER.

As discussed above, fluoroquinolones do not represent a clean experiment because of their interaction with the SOS system and the possible activity against nucleotide excision repair. Therefore, we have performed the requested experiments, and the resulting data show a combinatorial activity of the drugs and 4NQO. The three lead compounds show clear combinatorial effects with 4NQO, and this additional information has been made available in the supplementary material and referred to in the main manuscript.

2. As stated earlier, humans do not possess UvrA. There is a functional conservation of NER but no structural similarities exist (Petit & Sancar, 1999). Other (but not all) DNA repair pathways in bacteria do have human counterparts with structural conservation, which is why inhibiting NER is so attractive.
I recognize that there's no structural homolog in humans. While inhibiting an activity in vitro might be straightforward, proving specificity can be more challenging. For instance, urea can inhibit almost any protein in vitro but isn't a specifically good inhibitor. What experiments have the authors conducted to demonstrate specificity in vitro? It is essential to show that these inhibitors don't inhibit the ATPase activity of unrelated proteins in my opinion.

We have addressed this comment as suggested by the editor in point 3.

3. There is a problem of copy and paste with fig2c western blot as I already mentioned in my previous review.

We would like to point that figure 2c is not a western blot, our manuscript doesn`t contain any such experiment. However, figure 2c lacked the labels as suggested by reviewer 1 in the previous review and we have addressed the issue in the new version of the manuscript.

Reviewer #3: In my opinion, the authors have satisfactorily answered all comments. The use of a clinically relevant E. coli strain is a plus for this study.
Regarding UvrA homologues in human cells and the possibility of the existence of a human UvrA homologue, the NER machinery in human cells involves a multitude of proteins (about 30 factors), which act in complexes. For a review: <https://www.nature.com/articles/nrm3822>
None of these factors show homology with E. coli UvrA, the closest sequence homologue (25% homology) to uvrA is an ABC transporter of insulin because of its ATPase domain.
XPC is the main initiator of NER, except at UV lesions where the lesion is recognized by DDB.
Regarding DDB: “DDB may play a special role in the repair of UV damage, but it cannot be the sole damage recognition subunit of human excision nuclease” PMID: 8407968
“physical interaction between DDB and XPA plays an important role in the DDB-mediated NER reaction” PMID: 19056823
More importantly, the tested drugs have been already FDA-approved, which means that their innocuity for human cells has already been established. This is one of the advantages of drug repurposing, where drugs that are already used in humans would be used for another purpose. The combination of cisplatin and the selected drugs has not been tested yet but this would be, in my opinion, more relevant for clinical studies, rather than the proof of concept of potentiation of antimicrobial action of the combination, as shown here.

We want to thank the reviewer for the positive feedback.

**References**

Bhattacharya, Prabuddha, Srasta Mukherjee, and Santi M. Mandal. 2020. “Fluoroquinolone Antibiotics Show Genotoxic Effect through DNA-Binding and Oxidative Damage.” *Spectrochimica Acta. Part A, Molecular and Biomolecular Spectroscopy* 227 (February): 117634.

Crawford, Jeffrey, David C. Dale, and Gary H. Lyman. 2004. “Chemotherapy-Induced Neutropenia: Risks, Consequences, and New Directions for Its Management.” *Cancer* 100 (2): 228–37.

Curtin, J. P., W. J. Hoskins, S. C. Rubin, W. B. Jones, T. B. Hakes, M. M. Markman, B. Reichman, L. Almadrones, and J. L. Lewis Jr. 1991. “Chemotherapy-Induced Neutropenia and Fever in Patients Receiving Cisplatin-Based Chemotherapy for Ovarian Malignancy.” *Gynecologic Oncology* 40 (1): 17–20.

McDaniel, L. S., L. H. Rogers, and W. E. Hill. 1978. “Survival of Recombination-Deficient Mutants of Escherichia Coli during Incubation with Nalidixic Acid.” *Journal of Bacteriology* 134 (3): 1195–98.

Merschsundermann, V., K. Hauff, P. Braun, W. Lu, and H. Hof. 1994. “DNA-Damage Caused by Antibiotic Drugs - Quinolones.” *International Journal of Oncology* 5 (4): 855–59.

Mok, Wendy W. K., and Mark P. Brynildsen. 2018. “Timing of DNA Damage Responses Impacts Persistence to Fluoroquinolones.” *Proceedings of the National Academy of Sciences of the United States of America* 115 (27): E6301–9.
